# Chronic inflammatory demyelinating polyneuropathy‐like neuropathy in IgG4‐related disease

**DOI:** 10.1111/cns.13747

**Published:** 2021-10-25

**Authors:** Cong‐Cong Wang, Bin Liu, Xiao‐Li Li, Bing Yang, Yan‐Bin Li, Rui‐Sheng Duan

**Affiliations:** ^1^ Department of Neurology The First Affiliated Hospital of Shandong First Medical University Jinan China; ^2^ Shandong Institute of Neuroimmunology Jinan China

**Keywords:** Chronic inflammatory demyelinating polyneuropathy, IgG4‐related disease, mechanism, peripheral neuropathy

## CONFLICT OF INTEREST

The authors declare no conflict of interest.

## INFORMED CONSENT

We have obtained the patient's permission and informed consent for the publishing of his information and images.

Dear Editor,

Chronic inflammatory demyelinating polyneuropathy (CIDP) is an acquired immune‐mediated disorder of peripheral neuropathy characterized by chronic progressive or relapsing motor and sensory deficits.^1^ IgG4‐related disease (IgG4‐RD) is a systemic fibroinflammatory disorder characterized by organ enlargement, an elevated serum IgG4 level, and characteristic histopathologic features.^2^ IgG4‐related disease can affect multiple organ systems, including the central and peripheral nervous systems,^3^ and it may initially manifest with neurological symptoms only. Here, we firstly report the CIDP‐like neuropathy in IgG4‐RD.

A 55‐year‐old immunocompetent man with history of left exophthalmia for 5 years was diagnosed as orbital inflammatory pseudotumor and has been receiving oral prednisone for half a year. Two months ago, after stopping oral prednisone, the patient presented with weakness and numbness of limbs. The patient had no medical history for diabetes, metabolic, toxic, or any neurological diseases. Neurological examination revealed bilateral upper limbs weakness (5/5 shoulder abduction and 3/5 fingers abduction), bilateral lower limbs weakness (3/5 knee flexion, 4/5 knee extension, 5/5 dorsiflexion, and 5/5 toe extension), and decreased sensation of vibration in the upper and lower extremities. On both sides, the tendon reflexes were diminished in all extremities.

The serum IgG and IgE levels increased (IgG, 2060 mg/dl [reference range, 700–1600 mg/dL]; IgE, 134 IU/ml [reference range, <10 IU/L]). Additionally, the serum IgG4 level was elevated (1550 mg/dl [reference range, 3‐201mg/dl]). A serum autoantibody test was positive for antinuclear antibody (titer, 1:100) and anti‐SS‐A antibodies, but negative for anti‐SS‐B antibodies, anti‐DNA antibodies, and antiphospholipid antibodies. The test was also negative for anti‐neutrophils cytoplasmic antibodies and M‐protein. Autoantibodies anti‐ganglioside antibodies and paranodal proteins such as neurofascin (NF) 155, NF186, contactin‐1 (CNTN1), contactin‐2 (CNTN2), and contactin‐associated protein 1 (Caspr1) were negative. Cerebrospinal fluid (CSF) protein level (81 mg/dl) was found, and there was no leukocyte in CSF. Gastrointestinal endoscope showed no evidence of malignant tumor in the upper and lower gastrointestinal tracts. Findings on chest computed tomographic and abdominal ultrasound, and magnetic resonance imaging scans of the cranial spinal cord did not show any apparent abnormalities.

**FIGURE 1:**
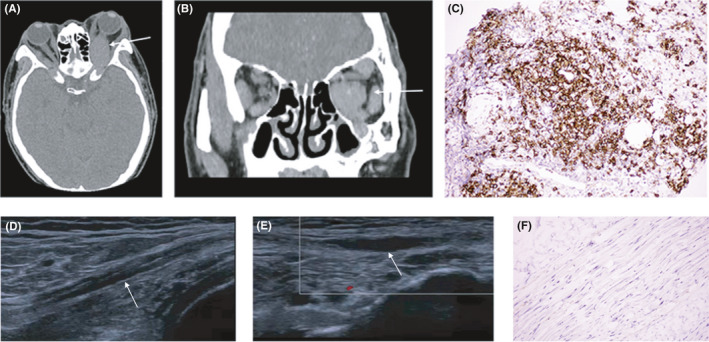
Orbital imaging features and nerve ultrasound photographs. Left optic nerve and orbital muscle can be detected in the lesion in axial (A) and coronal (B) imaging. (*arrow*). Marked infiltration of IgG4‐positive plasma cells is shown (C). Nerve segmental enlargement was shown of right peroneal nerve bundle (*arrow)* (D) and left peroneal nerve bundle(*arrow*) (E). A perineural IgG4‐positive plasma cell infiltration was not observed (F) (original magnification ×200)

Enhanced computed tomography scan showed an enlargement of the left optic nerve and orbital muscles (Figures 1A‐B). The orbital biopsy showed an increased number of IgG4‐positive plasma cells constituting at least 50% of total IgG plasma cells and more than 30 IgG4^+^ plasma cells per high‐power field (Figures 1C). Nerve conduction tests showed prominent demyelinating patterns in the ulnar, femoral, and tibial nerves, such as reduced motor conduction velocities, partial motor conduction block, and prolonged distal latencies (Table 1), which met the European Federation of Neurological Societies/Peripheral Nerve Society electrophysiological criteria for definite CIDP. Prolongation of F‐wave latency showed in the ulnar, femoral, and tibial nerves. Additionally, the sensory conduction velocities were decreased in the median nerves, whereas the sensory nerve action potentials were normal. Nerve ultrasound showed significant thickening of bilateral brachial plexus nerves and uneven thickening of the sciatic nerve, tibial nerve, and common peroneal nerve bundles. (Figures 1D‐E). A sural nerve biopsy was unremarkable without IgG4‐positive plasma cell infiltration, and no demyelination was observed by Weil's hematoxylin myelin staining (Figures 1F). The patient was diagnosed as IgG4‐RD and received methylprednisolone treatment venously (80mg/d) for 2 weeks, followed by oral prednisolone (40mg/d) tapering schedule. His symptoms of weakness and numbness of limbs were improved gradually, and the IgG4 level was decreased to 113 mg/dl (vs 1550 mg/dl before treatment) after 4 months of methylprednisolone treatment.

**TABLE 1:** Nerve conduction study results

Nerve	Patient (R)	Patient (L)	Normal values
Median nerve			
DL (ms)	3.7	4.2	<4.0
MCV (m/s)	43.7	59	>50
CMAP(mV)	6.1	5.0	>5
F‐wave latency (ms)	32.6	31.7	<31
SCV (m/s)	39.2	42.2	>44
SNCP(μV)	13.5	17	>10
Ulnar nerve			
DL (ms)	3.0	2.4	<4.5
MCV (m/s)	37.4	35.4	>50
CMAP(mV)	2.7	4.7	>6
F‐wave latency (ms)	37.6	34	<32
SCV (m/s)	51.4	50.6	>44
SNCP(μV)	8.6	7.0	>8.5
Tibial nerve			
DL (ms)	5.6	4.7	<5.8
MCV (m/s)	36.1	35.4	>37
CMAP(mV)	8.4	5.0	>4.8
F‐wave latency (ms)	60.7	60.4	<58
Sural nerve			
SCV (m/s)	40.2	45.5	>41
SNCP(μV)	7.9	8.1	>6

Abbreviations: CMAP, compound muscle action potential; DL, distal latency; L, left; MCV, motor conduction velocity; R, right; SCV, sensory conduction velocity; SNAP, sensory nerve action potential.

Both the central and peripheral nervous systems can be involved in IgG4‐RD. IgG4‐related peripheral neuropathy has been reported,^4^ in which infiltration of IgG4‐positive plasma cells and fibrosis within the epineurium was observed, resulting in varying degrees of axonal damage with or without demyelination.^3^ In contrast, nerve conduction test in our patient demonstrates a prominent demyelinating pattern, without infiltration of IgG4‐positive plasma cells in sural nerve biopsy. Clinical manifestations, electrophysiological studies, CSF findings, and response to corticosteroid therapy resembled CIDP. It should be distinguished from CIDP mediated by IgG4 autoantibodies against nodal and paranodal proteins,^5^ but the patient has negative autoantibodies against paranodal proteins in our case.

To the best of our knowledge, this is the first case report that describes the clinical features of CIDP‐like neuropathy in IgG4‐RD, expanding the neurological phenotype for this rare disease. The pathogenesis of CIDP‐like neuropathy in IgG4‐RD is currently unknown. We hypothesize that it mimics the CIDP‐like neuropathy in IgG or IgA monoclonal gammopathy of undetermined significance (MGUS).^6^ Although not having M‐protein, the patient had higher levels of IgG and IgG4, suggesting that CIDP‐like neuropathy in IgG4‐RD and IgG MGUS may share similar immunological mechanisms.

## Data Availability

Data available on request from the authors.
